# Moments and momentum in the returns of securitized real estate: A cross-country study of risk factors driving real estate investment trusts before and during COVID-19

**DOI:** 10.1016/j.heliyon.2023.e18476

**Published:** 2023-07-20

**Authors:** Wendi Zhang, Bin Li, Eduardo Roca

**Affiliations:** Department of Accounting, Finance and Economics, Griffith Business School, Griffith University, Nathan Campus, Brisbane, Queensland, Australia

**Keywords:** REITs, Asset pricing model, Momentum, Moments

## Abstract

A real estate investment trust (REIT) is a company running a funding pool that allows people to invest in real estate without physical purchase. Since REITs are stock market-traded real estate assets, there is debate as to whether their returns are driven by stock market risk factors. In this regard, this paper examines the impact of the well-established equity market risk factors of momentum, skewness, and kurtosis on the returns of different types of REITs, including mortgage REITs (MREIT), equity REITs (EREIT), and hybrid REITs (HREIT), across five countries—Australia, the UK, the US, Japan, and Canada—during the period 2000–2022, controlling for other well-established factors in the asset pricing literature. The study first adds the skewness and kurtosis to analyze cross-national REIT returns via the Fama–French five-factor model. Next, the cross-national REIT dataset is built for the different periods and then tested for the robustness of the effect of the factors during the COVID-19 period. Findings indicate that the influence of momentum on the return of the REITs is consistently positive across countries and different types of REITs. However, the significance of momentum for different REITs in different countries varies. These results were robust during the COVID-19 period, providing further confirmation that REITs behave less like stocks rather than real estate investments, with significant implications for investors.

## 1 Introduction

Property investments provide investors with portfolio diversification benefits and a hedge against inflation. Being an exchange-traded form of property investment, REITs overcome issues of relative illiquidity, large investment requirements, and transaction costs inherent in the ownership of direct property assets. For REITs to be effective substitutes for real estate investments, they should demonstrate similar attributes to property and react similarly to market forces. Yet the fact that REITs are different from direct real estate may inhibit their ability to proxy for it. Since REITs are market-traded, it is claimed that they behave more like stocks and less like real estate. Hence, it is important to examine factors that impact REIT returns as distinct from both the stock and real estate markets, as previous research has not reached a consensus on the influence of different factors that may impact the returns of REITs.

The COVID-19 pandemic broke out in late 2019, affecting many aspects of life across most of the world. Governments introduced life-saving restrictive policies, such as closing cities and public areas, which also limited economic activities. S&P Report (2020) cautioned that this decline in economic activity might cause liquidity problems, resulting in a massive debt default and a new financial crisis. As predicted, the US stock market value lost 30% in one month in the early period of the pandemic due to the market panic. However, stimulated by governments' accommodative financial and monetary policies, financial markets have not just quickly recovered but have reached an unprecedented height. The REIT showed substantial resistance to risk in the quick U-shape recovery process, even though the initial drawdown of both Australian and UK REITs was more than 20%.

The history of REITs dates back to the middle of the 20th century. In 1960, in the process of amending the Cigar Excise Tax Extension, the United States Congress authorized the formation of REITs, which allowed companies in the real estate sector to pay REITs taxable exemption dividend [1]. Since the US was the first to establish REIT rules, other countries that went on to establish REITs drew on US provisions.

This paper examines the effect of momentum, kurtosis, and skewness on REIT returns, controlling for other factors that impact the returns. Although these factors are well-established in the asset pricing literature about stock markets, no study has yet examined them collectively in the context of the REITs market. We therefore conducted this study in the cross-national context of the REITs markets of Australia, the UK, the US, Japan, and Canada, examining different types of REITs from 2000 to 2022. The next section outlines why these countries provide an excellent laboratory for this investigation. In addition, in order to explain REIT risk resistance and compare the impact of the risk factors on REIT, this paper splits the data at the COVID outbreak, that is, pre-COVID (2000–2019) and COVID (2019–now) apart from using full-sample data, to check the robustness of the results.

The paper applies the extended Fama–French five-factor model that includes SMB (Size effect), HML (Value premium), momentum, skewness, and kurtosis factors to daily returns in all five countries' REIT markets against the daily return. Doan et al. [2] pointed out that both skewness and kurtosis explain Australian stock returns and consistently influence US stock returns. Other studies have employed a four-moment capital asset pricing model (CAPM) to estimate risk premium, and the MHAR-RV (Multivariate heterogeneous autoregressive-realized volatility) model to capture the long memory of REIT market variance [3,4]. However, no previous studies have yet analyzed the REIT market using the Fama–French five-factor model. Using this model, our study shows a different result from previous research, in that skewness has a more significant impact on REIT than kurtosis.

Furthermore, our research builds a cross-national dataset from five countries (Australia, the UK, the US, Japan, and Canada) in two different periods, pre-COVID and COVID. Since the COVID outbreak at the end of 2019, many researchers have attempted to discover its impacts on the financial market. Shu et al. (2021) found that COVID crashed the US stock market in the short term, and Hassan and Riveros [5] confirmed that COVID has had a negative short-term impact on the return of the stock market and Brent crude oil. However, few previous studies have so far attempted to explore COVID's effect on REIT [6]. Our results show that COVID has had only a slight impact on REITs due to aggressive financial and monetary policies; this will be explained in detail in the discussion section. We also confirm that REITs have a solid capacity for reducing contingency risk.

Finally, findings show that the influence of momentum on the return of REITs is mostly positive for different types of REITs in different countries, including EREITs, MREITs, and HREITs, which have not been examined by previous researchers. In comparison with momentum, skewness and kurtosis have a more negligible effect, and skewness shows more influence than kurtosis on REIT.

## 2 Institutional background

### 2.1 REIT

The abbreviation REIT stands for real estate investment trust, which is an open-end trust managed by a company that enables individuals to invest in real estate without physically purchasing properties. The first REIT was established by the US Congress, accompanied by four main regulations that aim to safeguard investors and govern the operations of REITs. These regulations include: (1) a requirement for REITs to possess at least 75% of their assets in real estate, government bonds, and cash, (2) a mandate for REITs to have more than 50% of the total shares owned by a combination of five or more individuals, (3) a condition where over 75% of the income must come from rental or sale of real estate, or from mortgage interest, and (4) an obligation for REITs to distribute at least 95% of their taxable income as dividends annually [7].

Based on their specific characteristics, REITs can be classified into three types: equity-REIT (EREIT), mortgage-REIT (MREIT), and hybrid-REIT (HREIT). EREITs are publicly traded companies primarily engaged in acquiring, managing, renovating, maintaining, and occasionally selling real estate properties. MREITs issue and hold debt instruments, such as loans, backed by real estate, and typically offer higher dividend yields than EREITs. On the other hand, HREITs combine the features and operations of both EREITs and MREITs [8].

Furthermore, even REITs have similar trading method with normal stocks, distinguishing between REITs and common stocks has been a challenge, but a number of studies [[9], [10], [11]] have highlighted key differences. REITs offer diverse subsectors within the real estate industry, each reacting differently to economic and market conditions, providing a unique analytical environment absent in the broader stock market. Additionally, REITs are structured to distribute a significant portion of their income as dividends, making them attractive to certain investors and influencing their pricing dynamics. Furthermore, REITs provide investors with accessibility to real estate investments without the need for substantial capital outlays required for direct property ownership, resulting in potentially different responses to market forces compared to general equities. Thus, these factors establish a distinct separation between REITs and common stocks.

### 2.2 The world REIT cross countries and times

The world's total real estate value was over $35 trillion, while the value of the world GDP (gross domestic product) was about $78 trillion in 2021; the value of the REIT market was over $2375 billion, and particulary the emerging market developed rapidly [12]. Table 1 shows that selected five countries own a primary REIT market of 383 listed REITs, which while being around 41.45% of global REITs, accounts for 85.64% of global REIT market capitalization. In contrast, the emerging market accounts for only 4% of global capitalization, although it has 326 REITs and 28.46% of the world commercial real estate (CRE). This lag indicates that REITs still have great potential for growth in the future.

**Table 1:** Overview of five countries and global market REIT.

	US	UK	Australia	Japan	Canada	Emerging market	Global market
GDP ($ Billion)	22,939.58	3108.42	1610.56	5103.11	2015.98	32,639.24	87,574.22
Percent of Global Market	26.19%	3.55%	1.84%	5.83%	2.30%	37.27%	100%
Number of listed REITs	185	53	41	63	41	326	924
Percent of Global Market	20.02%	5.74%	4.44%	6.82%	4.44%	35.28%	100%
Total commercial real estate (CRE) ($ Billion)	9797.04	1636.33	662.68	2288.54	819.05	9569.73	33,619.95
Percent of Global Market	29.14%	4.87%	1.97%	6.81%	2.44%	28.46%	100%
REITs Market Cap ($ Billion)	1599.68	100.97	120.69	137.15	76.24	95.26	2375.86
Percent of Global Market	67.33%	4.25%	5.08%	5.77%	3.21%	4.01%	100%
Listed REIT/Stock market	3.81%	3.44%	6.84%	4.01%	2.72%	3.83%	3.81%
Listed REIT/Total CRE	16.58%	6.91%	19.10%	10.73%	11.12%	9.78%	11.78%

*Source:* EPRA World REIT Report (2022).

As mentioned, the REIT markets of Australia, the UK, the US, Japan, and Canada provide an excellent laboratory for our investigation. First, these markets represent different stages of REIT development. For example, Australia has the largest share of REITs in the overall stock market of all five countries, while Canada has the slowest REIT development of the five countries. Although Japanese and UK REITs were established later than Canada, their market capitalization and number of REITs far exceed Canada's. Ghosh and Sun [13] explained that the amount of CRE available in these countries is linked to a higher development of REITs. Second, the higher GDP of these countries may create more REITs; for example, the GDPs of the US, Japan, and the UK are ranked in the same order as their respective numbers of listed REITs. In the US and Australia, however, REITs prefer CRE. We also observe that the earlier a country established a REIT market, the greater the REITs' investment prefer CRE, such as in the US, Australia, and Canada. Finally, the five countries have the largest REIT markets in their respective geographic regions: Europe, Asia Pacific, and the Americas. They share the similar legal, restriction and tax exemptions background in their REIT markets. But they can be nuanced in terms of regulation. For example, there is no specific capital requirement for listed REITs in Australia, the US, and the UK, and the listing requirements for REITs in Japan and Canada are 100 million yen and 1 million dollars respectively. In terms of treatment of foreign investors, the U.S., Canada, and the U.K. do not impose any restrictions on foreign investors; Australia requires that foreign investors cannot hold more than 10% of the REIT's shares, while Japan completely prohibits foreign investors from participating in local REITs. In addition, the five countries have different tax policies on REIT profit distributions: Australia, the U.S. and Canada do not pay tax on REIT shares that are not redeemed by investors; Japan pays tax based on the percentage of shares held in the REIT; and the U.K. pays a 20% tax on each REIT distribution. Overall, their REITs have different characteristics, and examination of the risk factors allows consideration of the various effects of these factors on each country's REITs.

In early 2020, the outbreak of COVID-19 led to a significant disruption to the world economy. The world's equity market, including the major stock indexes— Dow Jones Industries Average, NYSE, NIKKEI, FTSE 100, and ASX 200—declined by 33% [14]. Van Dijk et al. [15] analyzed the difference between supply and demand in the CRE market, and their results indicate the extremely short liquidity in real estate markets: The apartment, industrial, office, and retail markets decreased by 14%, 14%, 18%, and 20%, respectively. The REIT indexes of the US, the UK, Japan, and Australia also dropped by 20–25% between Feburary and April of 2020. These governments then introduced aggressive fiscal and monetary policies to reduce the impact of COVID-19 on their economies and financial markets, such as lowered interest rates, fiscal support via loan and loan guarantees, and extending bond buyback [14]. As a result, until June of 2020, the real estate value and REIT markets quickly returned to their pre-COVID levels. As such, unlike the stock indexes, such as S&P 500 and Dow Jones Industrial Average, which took six months to complete a U-shaped recovery, it took only four months for the REIT markets to bounce back. Balemi et al. [16] highlighted a similar finding in the real estate market, arguing that high transaction costs, liquidity constraints, and loss aversion during the period constrained real estate values from overacting to contingency. This paper seeks to explain this phenomenon by comparing the various risk factors before and during the pandemic with the guidance from finance theory.

## 3 Literature review and hypotheses

Previous studies undertaken on the factors affecting REIT returns includes investigations into volatility, liquidity, skewness, and kurtosis. Researcher indicated that those variables are important to predict the stocks and REITs return and future performance. For example, Fei et al. [17] analyzed the returns of REITs from 1987 to 2008 and found that volatility is essential in estimating the returns of REIT portfolios. Hung and Glascock [18] uncovered a positive relationship between volatility and momentum in the return of REITs, and Derwall et al. [19] observed a strong momentum effect in REIT returns. Likewise, Beracha and Skiba [20] confirmed that the momentum effect in home prices exists regardless of the period or geographical region. Both Hutson and Stevenson [21] and Xiong and Idzorek [22] found evidence of the effect of both skewness and kurtosis on REIT returns. Bhasin et al. [23] provided evidence of the influence of liquidity on REIT returns.

Factors such as skewness, kurtosis, and momentum have been proven to significantly impact REIT returns. However, most researchers have only employed one or two of these variables, and there are few studies that integrate them. For instance, Ayadi et al. [24] developed a model to estimate the influence of these variables, but their model is quite complex due to the large number of factors included, potentially leading to overfitting issues. Mansur et al. [25] found that REIT index returns can be explained by systematic skewness and other economic factors. Notably, even when they applied the economic factors as dummy variables, the effect of skewness remained significant. Jensen and Turner [26] constructed a multifactor regression model with different momenta on US REIT returns, and their results validated a strong relationship between momentum and REIT returns. These aforementioned studies each use different independent variables to analyze REIT returns. In our study, we consolidate the factors with significant impacts on REITs from the prior literature, such as skewness, kurtosis, momentum, and apply a multifactor regression model to analyze REIT returns in five countries over time.

### 3.1 Momentum

Momentum in finance could be defined as the rate of acceleration of asset's price, in other words, momentum is the speed at which the price is changing [27]. Since the introduction of momentum, many researchers and investors have realized the importance of momentum in academic and investment terms. For instance, Carhart [28] attempted to capture the momentum effect in a four-factor capital asset pricing model (CAPM) via the difference between returns on the monthly diversified portfolio of the winners and losers. The momentum strategy, which involves buying the past three to twelve months' winner stocks and selling the past loser stocks, was shown to deliver positive excess returns in the US stock market. Subrahmanyam [29] further showed that momentum is a robust risk factor in the Fama–French four-factor model and is positively related to excess stock return. Momentum strategy was involved in building multi-assets portfolios and boosting the annual Sharpe ratio from 0.45 to 0.9 [30]. However, there are controversies about the relationship between momentum and assets, firstly, on the choice of the interval of momentum, and secondly, on the positive or negative correlation between momentum and the future income of assets. For example, Baltzer et al. [31] reported the negative coefficient between the lagged momentum trading with returns in German market; but Lim et al. [32] found the momentum positive related to S&P 500 returns in the US market. Thus, we try to identify how the momentum effect the different REIT market. The first hypothesis is stated as follows.

Hypothesis 1 The momentum of returns of REITs positively affects REITs' expected returns.

### 3.2 Skewness

Skewness is a crucial statistical measure that captures the asymmetry of a probability distribution about its mean. Early literature on skewness and finance dates back to 1976, Kraus and Litzenberger [33] pointed out that investors should choose the positive skewness, also implying assets with greater skewness might command higher prices and, consequently, lower expected returns. Their findings provided a unique perspective on how skewness could affect expected stock returns. Their study also laid the foundation for later research on skewness, including stock and REIT market.

For example, co-skewness has been proven to have a significant impact on deciding the weighting factors in portfolio optimization [34]. Furthermore, Chen et al. [35] added skewness as a higher moment in the CAPM to estimate the excess return of the Chinese stock market. Their study suggests that skewness affects CAPM, individual security, and even portfolio investment. Glascock et al. [36] found that the skewness of REIT returns is significantly negative, which could be due to the unique characteristics of real estate assets and market conditions. However, the systematic skewness showed different statistical and financial significant in different markets. For instance, the market skewness premium in the S&P 500 was statistically significant but could not explain the stock returns [37], in contrast, Jiang et al. [38] reported that the systematic skewness has a significant negative association with stock returns. Therefore, we attempt to identify the systematic skewness effect in different countries’ REIT market and different types of REITs.

Hypothesis 2 Systematic skewness is positively related to the expected returns of REITs.

### 3.3 Kurtosis

Kurtosis is similar to skewness in that it is a significant independent risk factor and systematic variance in studying excess stock returns. In finance, kurtosis measures the risk of extreme outcomes, high kurtosis investment usually produces both positive and negative results. Scott and Horvath [39] were among the pioneers to underscore the importance of kurtosis in option pricing, paving the way for further research on the influence of kurtosis on asset returns. Fang [40] used kurtosis and skewness to explain the asset return distribution in the US stock market. Hwang and Satchell [41] studied an emerging stock market using a higher moment CAPM that includes kurtosis and skewness; their results showed that a higher moment CAPM has better performance than the traditional CAPM. Also, kurtosis has been used as a dependent variable in recent years in the analysis of stocks by fintech, the results showed that kurtosis can improve the prediction accuracy of stock return volatility in mid- and long-term. Xu and Shang [42] observed the corresponding relationship between the kurtosis and stock returns in Asian and North American market, this connection directly related to the degree of stability of financial market. They claimed that kurtosis will be an important independent variable in stock and financial market analysis. In our research, we suppose the kurtosis has positive relationship with REIT returns in five countries.

Hypothesis 3 Kurtosis of REIT returns is positively related to the expected returns of REITs.

## 4 Methodology

### 4.1 Model

This paper examines the effect of SMB, HML, momentum, kurtosis, and skewness on REIT returns. To identify the impacts of these factors on the return of REITs, we have extended the Fama–French model; the model is stated as follows:

(1) $$R_{t}-R_{f,t}=\alpha +\beta_{1}\left({Rm_{t}-Rf}_{t}\right)+\beta_{2}SMB_{t}+\beta_{3}HML_{t}+\beta_{4}Momentum_{t}+\beta_{5}Skewness_{t}+\beta_{6}{Kurtosis}_{t}+\varepsilon_{t}$$

Equation (1) indicates that $R_{f}$ is the risk-free rate and $R_{m}$ refers to the daily market return. The other three variables, momentum, skewness, and kurtosis, are the REITs’ momentum, skewness, and kurtosis in the selected period. In addition, α is the intercept of the regression analysis, and $\beta_{i},i=1,\ldots ,6$ are the coefficients of these variables; ε is an error term.

### 4.2 Sample and data

Daily data were obtained for five countries—Australia, the UK, the United States, Japan, and Canada—for different types of REITs, including MREITs, EREITs, and HREITs, for the period 2000–2022. EREITs are made up of profitable real estate portfolios, directly creating profits in their daily operations—for example, by being rented to others or sold to other REITs to create sales [43]. The main feature of EREITs is that their assets can create profits in daily operations; investors share the taxable incomes created by assets in business operations as dividends [13]. MREITs differ from EREITs because their investment objects are mortgage-backed securities or mortgages [44]. In addition, assets from MREITs do not create profits in business operations because they are not involved in daily operations. Finally, HREITs combine the other two REIT categories, so that the assets of HREITs may contain real estate properties and mortgage-backed securities [45]. The test results are split into three periods, pre-COVID, COVID, and the full sample, to check the robustness of the results.

The daily data used in this research was collected from Bloomberg, Investing.com, and Yahoo! Finance. The UK built the REIT market in 2007. For those countries that had not established their REIT markets before 2000, the commencement year of the dataset is the first year of their REIT markets. The main stock returns from each country are used to calculate the systematic skewness and kurtosis, and the composites of those indexes are used to calculate the SMB and HML. The formular used to calculate SMB and HML is stated as follow [46]:

(2) SMB=(S/H+S/M+S/L)/3－(B/H+B/M+B/L)/3

(3) HML=(S/H+B/H)/2－(S/L+B/L)/2

In Equations (2), (3)), the stocks are allocated into 2 groups according to market value, small (S) and big (B). Low (L), medium (M), and high (H) are ranked based on stocks book values. We calculate the momentum with t-1 time scale.

## 5 Empirical results

### 5.1 Descriptive statistics

The descriptive statistics of the values of these factors are presented in Table 2, including their average value (Avg), standard error (Std Err), median, and standard deviation (Std Dev), sample variance (Var), minimum value (Min), and maximum value (Max). The descriptive statistics analysis indicates significant gaps in REIT performance in the five countries studied.

**Table 2:** The descriptive statistics of factors.

Australia							
	Avg	Std Err	Med	Std Dev	Var	Min	Max
R	0.002	0.003	0.005	0.043	0.002	−0.253	0.141
Rf	0.046	0.001	0.052	0.014	0.000	0.019	0.072
R–Rf	−0.044	0.003	−0.042	0.046	0.002	−0.305	0.086
Rm	0.003	0.003	0.007	0.046	0.002	−0.213	0.132
Rm-Rf	−0.043	0.003	−0.040	0.048	0.002	−0.257	0.085
RS	0.006	0.005	0.008	0.078	0.006	−0.319	0.238
RB	0.013	0.006	0.014	0.098	0.010	−0.404	0.294
RL	0.004	0.002	0.008	0.036	0.001	−0.117	0.075
RH	0.004	0.002	0.006	0.036	0.001	−0.104	0.076
SMB	−0.006	0.004	−0.006	0.058	0.003	−0.251	0.181
HML	0.000	0.000	0.000	0.003	0.000	−0.006	0.015
Momentum	0.038	0.134	0.245	2.040	4.161	−11.543	5.557
Skewness	−0.025	0.037	−0.046	0.550	0.302	−1.616	2.146
Kurtosis	−0.363	0.061	−0.552	0.915	0.837	−1.623	4.316
**Canada**							
	Avg	Std Err	Med	Std Dev	Var	Min	Max
R	0.004	0.003	0.008	0.041	0.002	−0.223	0.140
Rf	0.034	0.001	0.034	0.014	0.000	0.010	0.065
R–Rf	−0.029	0.003	−0.024	0.043	0.002	−0.261	0.106
Rm	0.004	0.003	0.008	0.040	0.002	−0.169	0.118
Rm-Rf	−0.030	0.003	−0.023	0.042	0.002	−0.207	0.078
RS	0.003	0.003	0.004	0.051	0.003	−0.241	0.140
RB	0.004	0.003	0.009	0.045	0.002	−0.188	0.184
RL	0.000	0.005	0.000	0.075	0.006	−0.353	0.161
RH	0.004	0.002	0.008	0.037	0.001	−0.164	0.119
SMB	−0.001	0.002	−0.002	0.023	0.001	−0.097	0.082
HML	0.004	0.004	0.006	0.055	0.003	−0.142	0.189
Momentum	0.014	0.011	0.030	0.169	0.029	−0.893	0.471
Skewness	−0.079	0.051	−0.026	0.751	0.564	−2.530	3.020
Kurtosis	0.807	0.127	0.232	1.869	3.494	−1.252	12.051
**Japan**							
	Avg	Std Err	Med	Std Dev	Var	Min	Max
R	0.004	0.004	0.007	0.052	0.003	−0.237	0.241
Rf	0.010	0.000	0.012	0.006	0.000	−0.002	0.019
R–Rf	−0.005	0.004	−0.002	0.053	0.003	−0.252	0.236
Rm	0.002	0.004	0.006	0.055	0.003	−0.238	0.128
Rm-Rf	−0.008	0.004	−0.004	0.056	0.003	−0.253	0.116
RS	0.004	0.005	0.010	0.056	0.003	−0.199	0.168
RB	0.002	0.004	0.007	0.052	0.003	−0.203	0.121
RL	0.003	0.004	0.007	0.050	0.002	−0.149	0.099
RH	0.006	0.004	0.008	0.043	0.002	−0.128	0.091
SMB	0.002	0.002	0.004	0.023	0.001	−0.069	0.060
HML	−0.002	0.002	−0.002	0.020	0.000	−0.059	0.063
Momentum	0.136	0.182	0.364	2.505	6.274	−10.48	10.651
Skewness	0.092	0.052	0.045	0.724	0.524	−1.870	1.923
Kurtosis	0.726	0.117	0.328	1.613	2.600	−1.432	6.090
**UK**							
	Avg	Std Err	Med	Std Dev	Var	Min	Max
R	−0.001	0.005	0.000	0.061	0.004	−0.225	0.237
Rf	0.034	0.001	0.037	0.015	0.000	0.006	0.057
R–Rf	−0.029	0.005	−0.023	0.064	0.004	−0.262	0.202
Rm	0.004	0.003	0.009	0.037	0.001	−0.134	0.092
Rm-Rf	−0.027	0.003	−0.022	0.040	0.002	−0.178	0.057
RS	0.006	0.003	0.010	0.045	0.002	−0.201	0.259
RB	0.003	0.003	0.009	0.038	0.001	−0.119	0.121
RL	0.002	0.003	0.009	0.044	0.002	−0.168	0.110
RH	0.005	0.003	0.009	0.040	0.002	−0.130	0.101
SMB	0.003	0.002	0.004	0.029	0.001	−0.086	0.138
HML	0.004	0.002	0.001	0.029	0.001	−0.079	0.129
Momentum	−0.332	0.424	0.007	5.275	27.82	−19.57	9.973
Skewness	−0.019	0.055	−0.054	0.595	0.354	−1.360	1.373
Kurtosis	−0.373	0.242	−0.003	2.598	6.749	−5.879	4.330
**US**							
	Avg	Std Err	Med	Std Dev	Var	Min	Max
R	0.069	0.060	0.009	0.882	0.777	−0.308	12.83
Rf	0.035	0.001	0.034	0.012	0.000	0.015	0.067
R–Rf	0.036	0.060	−0.022	0.881	0.777	−0.348	12.79
Rm	0.004	0.003	0.008	0.041	0.002	−0.141	0.106
Rm-Rf	−0.030	0.003	−0.026	0.043	0.002	−0.180	0.074
RS	0.008	0.004	0.013	0.052	0.003	−0.211	0.186
RB	0.006	0.003	0.010	0.040	0.002	−0.163	0.105
RL	0.008	0.003	0.012	0.049	0.002	−0.220	0.163
RH	0.006	0.003	0.011	0.041	0.002	−0.174	0.110
SMB	0.002	0.002	0.002	0.024	0.001	−0.056	0.104
HML	−0.002	0.001	−0.001	0.015	0.000	−0.067	0.046
Momentum	0.044	0.039	0.024	0.550	0.303	−2.023	6.152
Skewness	−0.063	0.038	−0.064	0.551	0.304	−1.402	1.526
Kurtosis	−0.326	0.123	−0.476	1.792	3.212	−5.554	4.447

Notes: R indicates the monthly return rate of these REITs, Rf indicates risk-free rate, R–Rf indicates the excess return rate, Rm indicates the market return, Rm-Rf indicates the market premium, RS indicates that the return of the small-cap portfolio, RB indicates the return of the big-cap portfolio, RL indicates the return of the low book value portfolio, and RH indicates the return of the high book value portfolio. SMB indicates the gaps between the returns of the small-cap portfolio and the big-cap portfolio, and HML indicates the gaps between the returns of the low book value portfolio and the high book value portfolio. The information shows that the average return of the REITs in these countries in the past decades was relatively low, except in the US market. At the same time, the momentums of these REITs are all positive, except in the UK market.

The US REITs have the highest average monthly returns. The average values of the excess returns of the REIT indexes in Australia, the UK, the US, Japan, and Canada are −4.43%, −2.85%, 3.64%, −0.51%, and −2.95%, respectively.

There was a notable change in the excess returns of the five stock markets before and after the 2008 Global Financial Crisis (GFC). This can be seen from the changes in the REIT indexes of the five countries, as the statistical information clearly illustrates. The average excess returns in the two years before2007^1^ were −0.0132 (Japan), −0.0427 (Australia), −0.0270 (Canada), and 0.5460 (the US), while the average excess returns in the two years after 2007 were 0.0979 (Australia), −0.0651 (Canada), −0.0174 (Japan), −0.0233 (the UK), −0.0001 (the US). Comparing the two groups of excess returns, it is clear that the 2008 GF C influenced the REIT markets in Australia, Canada, and the US, and that their average excess returns mainly decreased.

When the value of the momentum is negative, the reverse effect exists in the stock market. The mean values of the momentum for each of the five countries reveal that the momentum effect exists in all except the UK. The average skewness values of the countries' REIT returns, except for Japan's, are negative but are close to zero, suggesting that the selected monthly returns are almost skewed to the left. The average monthly kurtosis values of those in Australia, the UK, and the US are negative, suggesting that the REIT distributions in these three countries have lighter tails.

**Fig. 1:**
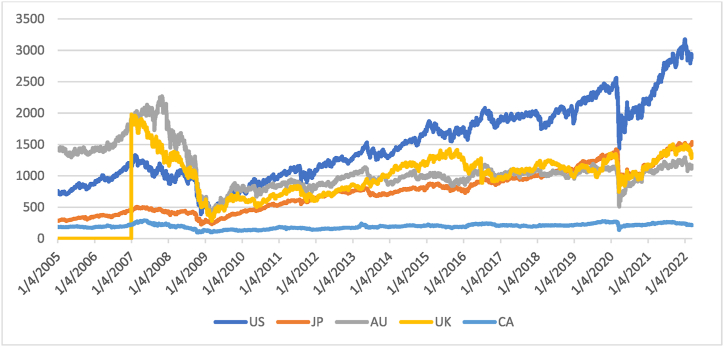
The movement of REIT index prices in the five countries, April 2005–April 2022.

**Fig. 2:**
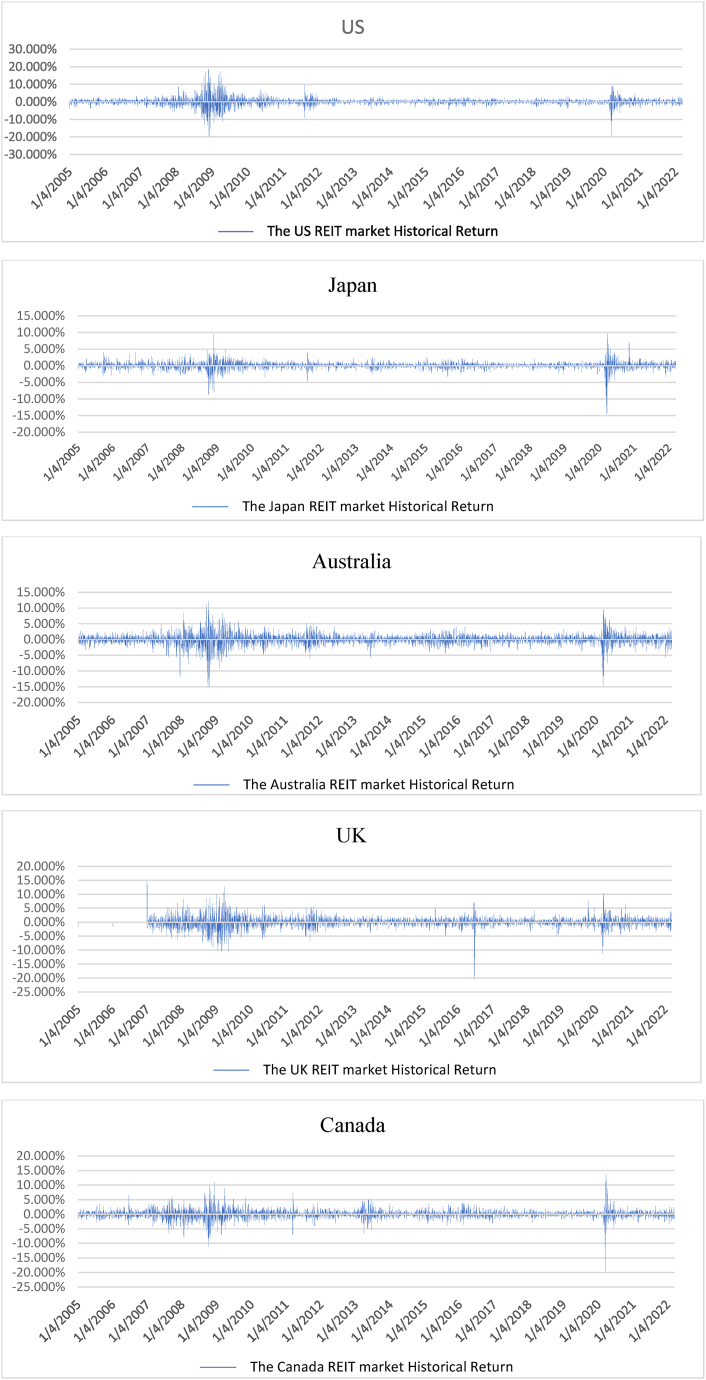
The movement of the REIT return indexes in the five countries.

Fig. 1 shows the movement of the prices of the REIT indexes. From 2007 to 2008, the GFC caused a decrease in all the REIT prices. It is easy to understand that this financial crisis impacted the real estate industry in these countries. Fig. 2 shows the returns of the REIT indexes in the five countries. Before 2007, their performances fluctuated slightly, but from 2007 to 2008, they continued to fluctuate and were almost negative; the GFC had severely impacted these REIT indexes. From 2009 to 2016, the returns of these REIT indexes both relatively and positively reflect that they are recovering from 2008.

### 5.2 Unit-root test results

A unit-root test, which evaluates the stationarity of a time series, is critical before running a multi-factor regression analysis as many econometric models require a dataset with constant mean, variance, and autocorrelation over time. Non-stationary data (containing a unit root) can lead to spurious regressions, where regression results, including estimated coefficients and standard errors, could be misleading and unreliable [47]. To check the stationarity of our variables, we performed three unit-root tests: the augmented Dickey–Fuller (ADF) test, the Phillips–Perron (PP) test, and the Dickey–Fuller-GLS (DF-GLS) test. The test results reported in Table 3 suggest that all variables are stationary.

**Table 3:** The unit-root test results.

Australia			
	ADF test	PP test	DF-GLS test
Rt-Rf	−11.936 (0.000)	−12.724 (0.000)	−4.258 (0.000)
Rm-Rf	−12.376 (0.000)	−12.727 (0.000)	−.3.363 (0.000)
SMB	−12.146 (0.000)	−14.793 (0.000)	−2.192 (0.000)
HML	−14.184 (0.000)	−14.166 (0.000)	−11.090 (0.000)
Momentum	−11.605 (0.000)	−11.844 (0.000)	−11.262 (0.000)
Skewness	−13.260 (0.000)	−13.299 (0.000)	−4.867 (0.000)
Kurtosis	−15.666 (0.000)	−15.675 (0.000)	−14.856 (0.000)
**UK**			
	ADF test	PP test	DF-GLS test
Rt-Rf	−11.591 (0.000)	−11.712 (0.000)	−2.723 (0.000)
Rm-Rf	−12.894 (0.000)	−13.061 (0.000)	−9.1 (0.000)
SMB	−12.553 (0.000)	−12.533 (0.000)	−10.371 (0.000)
HML	−13.551 (0.000)	−13.602 (0.000)	−1.758 (0.000)
Momentum	−12.103 (0.000)	−12.155 (0.000)	−9.014 (0.000)
Skewness	−10.081 (0.000)	−10.069 (0.000)	−4.297 (0.000)
Kurtosis	−10.830 (0.000)	−13.285 (0.000)	−9.954 (0.000)
**US**			
	ADF test	PP test	DF-GLS test
Rt-Rf	−14.500 (0.000)	−14.500 (0.000)	−14.499 (0.000)
Rm-Rf	−14.435 (0.000)	−14.449 (0.000)	−4.115 (0.000)
SMB	−15.818 (0.000)	−15.818 (0.000)	−11.179 (0.000)
HML	−14.849 (0.000)	−14.863 (0.000)	−10.378 (0.000)
Momentum	−13.462 (0.000)	−13.462 (0.000)	−13.438 (0.000)
Skewness	−14.793 (0.000)	−14.830 (0.000)	−11.886 (0.000)
Kurtosis	−13.649 (0.000)	−13.731 (0.000)	−12.089 (0.000)
**Japan**			
	ADF test	PP test	DF-GLS test
Rt-Rf	−10.902 (0.000)	−10.916 (0.000)	−9.46 (0.000)
Rm-Rf	−13.360 (0.000)	−13.400 (0.000)	−12.594 (0.000)
SMB	−13.475 (0.000)	−13.475 (0.000)	−4.206 (0.000)
HML	−11.262 (0.000)	−11.544 (0.000)	−10.238 (0.000)
Momentum	−10.531 (0.000)	−10.568 (0.000)	−9.884 (0.000)
Skewness	−13.042 (0.000)	−13.031 (0.000)	−8.174 (0.000)
Kurtosis	−15.065 (0.000)	−15.143 (0.000)	−12.32 (0.000)
**Canada**			
	ADF test	PP test	DF-GLS test
Rt-Rf	−12.396 (0.000)	−12.533 (0.000)	−5.162 (0.000)
Rm-Rf	−12.349 (0.000)	−12.453 (0.000)	−1.868 (0.000)
SMB	−14.434 (0.000)	−14.438 (0.000)	−11.124 (0.000)
HML	−10.274 (0.000)	−10.262 (0.000)	−10.047 (0.000)
Momentum	−12.834 (0.000)	−12.883 (0.000)	−12.815 (0.000)
Skewness	−14.756 (0.000)	−14.957 (0.000)	−2.273 (0.000)
Kurtosis	−13.350 (0.000)	−13.348 (0.000)	−4.193 (0.000)

### 5.3 Impact of Moments and momentum on general REITs

We conducted a regression analysis for the five countries for each of the three different periods: the full-period, pre-COVID, and COVID. Table 4 shows the results. We report the adjusted R-Square, t-statistics, and coefficient.

**Table 4:** The regression results of the full-period, pre-COVID, and COVID.

Full Period							
	AU	UK	US	US(Model_2)	US (Model_3)	Japan	Canada
Rm-Rf	0.018*** (0.003)	0.011*** (0.0025)	0.018*** (0.018)	−5.768*** (0.855)	−5.803*** (0.853)	0.002 (0.650)	0.023*** (0.0004)
SMB	0.412*** (0.0031)	0.349*** (0.00238)	−0.258*** (0.0004)	−5.754*** (1.449)		2.451*** (0.00)	0.041 (0.301)
HML	0.438*** (0.0051)	0.229*** (0.002)	−0.662*** (0.00399)		9.225*** (2.269)	2.321*** (0.00)	−0.03 (0.2522)
Momentum	0.499*** (0.00)	0.489*** (0.00)	0.495*** (0.00)	1.611*** (0.065)	1.616*** (0.065)	0.264*** (0.0031)	0.142*** (0.00174)
Skewness	0.001*** (0.0024)	0.000 (0.051)	0.000 (0.963)	0.066 (0.059)	0.06 (0.060)	0.003*** (0.0071)	0.009** (0.008)
Kurtosis	0.001 (0.287)	0.000 (0.347)	−0.000*** (0.6359)	−0.001 (0.018)	−0.003 (0.018)	−0.000 (0.0021)	−0.001 (0.0001)
R-Squared	0.494	0.482	0.785	0.768	0.769	0.725	0.896

Pre-COVID					
	AU	UK	US	Japan	Canada
Rm-Rf	0.018*** (0.016)	0.011*** (0.0025)	0.017*** (0.018)	0.002 (0.650)	0.023*** (0.0004)
SMB	0.410*** (0.00315)	0.349*** (0.00238)	−0.263*** (0.0004)	2.451*** (0.00)	0.04 (0.301)
HML	0.437*** (0.00512)	0.229*** (0.002)	−0.661*** (0.0004)	2.321*** (0.00)	−0.03 (0.2522)
Momentum	0.499*** (0.00)	0.489*** (0.00)	0.498*** (0.00)	0.264*** (0.0031)	0.142*** (0.00174)
Skewness	0.001*** (0.0024)	0.001 (0.051)	0.001 (0.963)	0.003*** (0.0071)	0.009** (0.008)
Kurtosis	0.001 (0.287)	0.000 (0.347)	−0.003 (0.6359)	−0.000 (0.0021)	−0.001 (0.0001)
R-Squared	0.494	0.481	0.785	0.725	0.897
COVID					
	AU	UK	US	Japan	Canada
Rm-Rf	0.018*** (0.016)	0.011*** (0.0025)	0.018*** (0.018)	0.002 (0.650)	0.023*** (0.0004)
SMB	0.401*** (0.0031)	0.349*** (0.00238)	−0.258*** (0.0004)	2.451*** (0.00)	0.039 (0.301)
HML	0.437*** (0.0051)	0.229*** (0.002)	−0.659*** (0.00399)	2.321*** (0.00)	−0.031 (0.2522)
Momentum	0.499*** (0.00)	0.489*** (0.00)	0.497*** (0.00)	0.264*** (0.0031)	0.142*** (0.00174)
Skewness	0.000*** (0.0024)	0.000 (0.051)	0.000 (0.963)	0.003*** (0.0071)	0.009** (0.008)
Kurtosis	0.000 (0.287)	0.000 (0.347)	−0.000 (0.6359)	−0.000 (0.0021)	−0.001 (0.0001)
R-Squared	0.494	0.482	0.785	0.725	0.897

Note: ***, **, and × represent that the results are statistically significant at the levels of 1%, 5%, and 10%.

Overall, the adjusted R-Squared values show a good fit for all countries and time frames. In addition, the p-values indicate that those factors can explain the REIT return. The effect of skewness and kurtosis on REIT is small in all countries. Furthermore, the results appear largely similar to the full-period results. This could suggest that the financial factors influencing the markets before COVID were consistent with the broader time frame. Uddin et al. [48] recognized a similar situation in the stock market; they argued that the pandemic crisis only creates volatility in the developed stock market in the short term.

Australian REIT is positive for all control variables, and all p-values of the control variables show statistical significance, except kurtosis. The R-squared value of this regression model is 0.494, which reveals that the regression model fits most of the samples well.

The excess return of the UK REITs is positively related to the excess return of all variables; even so, skewness and kurtosis hardly affected it. The R-Squared indicates that this regression model can explain about 48.2% of the variation of the returns. According to the p-values of these coefficients, only the coefficients of Rm-Rf, SMB, HML, and momentum are statistically significant, at the level of 5%.

The excess return of the US REITs is positively related to market excess return, momentum, and skewness, but is negatively related to HML, SMB, and kurtosis. The statistical information of the regression results shows that multicollinearity between SMB and HML does not affect the reliability of the regression model. The R-Squared values indicate that eliminating either SMB or HML from the original model would not affect the reliability. The p-values of the three regression models are all less than 0.001. In summary, multicollinearity does not affect the regression results of the model, so the original model was used for all the other countries.

The negative coefficient of the market premium in Table 4 for the US REIT markets (Models 2 and 3) could be explained by two reasons: the diversification of REIT and the US market conditions. First, REITs are generally considered as a good diversifier due to their low correlation with other asset classes. When the market premium is negative, it indicates a perfect diversification effect where the REIT's returns increase when the market returns decrease, and vice versa. This could potentially be the case for US REITs in Models 2 and 3. Second, the negative market premium could also be a result of market conditions during the period of the study. For instance, the market might have been gone through a downturn, and the REITs could have been performing relatively well due to their inherent characteristics, such as stable cash flows from rental income.

Furthermore, the contrasting result from Model 1 and Models 2 and 3 may be caused by the different specifications of models. Models 2 and 3 do not include the HML or SMB factor. When the SMB or HML factors, which do have a significant effect on REIT returns, are omitted, their effect may be incorrectly assigned to the Market Premium. This can cause a bias in the coefficient estimate of the market premium, making it larger or smaller than it should be. However, we could not find the previous research test HML or SMB independently, so further analysis and potentially additional data might be needed to definitively explain these different coefficients.

In the Japanese dataset, the excess return of the REITs is negatively related to kurtosis but positively related to the other four independent variables. The R-Squared value of the regression model is 0.725. Similarly, the excess return of the Canadian REITs is negatively related to kurtosis and HML but is positively related to the other variables. Canada had the highest R-Squared value, suggesting that the model explained the variance in the dependent variable exceptionally well for Canada.

These results indicate that the multicollinearity of the US samples does not influence the regression analysis. The cross-national regression results show that the regression model is reliable for illustrating the relationship between the excess return of the REITs and the six independent variables. They also show that the influence of momentum, skewness, and kurtosis on the excess return of the REITs is diverse. Therefore, further analysis is needed to judge these countries' unique situations and identify those variables’ influence on the excess return of the REITs in the different countries.

### 5.4 Influence of moments and momentum on the returns of EREITs, MREITs, and HREITs

To test the influence of the three variables on different types of REITs, we conducted a regression analysis of EREITs, MREITs, and HREITs by replacing the general returns of the REITs with the specific returns of each type. The regression results are shown in Table 5.

**Table 5:** The regression results of the EREITs, MREITs, and HREITs.

	EREITs	EREITs	MREITs	HREITs	HREITs
	UK	US (Model_2)	US (Model_3)	UK	US
Rm-Rf	0.234*** (0.037)	0.232*** (0.045)	0.034 (0.024)	0.261*** (0.043)	0.091*** (0.032)
SMB	0.058 (0.042)	0.253 (0.221)	0.009 (0.142)	0.116*** (0.043)	−0.014 (0.164)
HML	−0.039 (0.045)	−0.082 (0.346)	0.017 (0.237)	0.025 (0.051)	−0.157 (0.278)
Momentum	0.011*** (0.000)	0.108*** (0.004)	0.439*** (0.010)	0.018*** (0.001)	0.351*** (0.009)
Skewness	−0.001 (0.002)	−0.004 (0.003)	−0.002 (0.001)	−0.002 (0.002)	−0.002 (0.002)
Kurtosis	−0.0003 (0.000)	−0.0002 (0.001)	0.0002 (0.000)	−0.0003 (0.000)	0.0003 (0.000)
R-Squared	0.952	0.894	0.961	0.769	0.768

Note: ***, **, and × represent that the results are statistically significant at the levels of 1%, 5%, and 10%.

According to these regression results, in both the UK and US, EREITs show a significant positive correlation with the excess market return and Momentum, implying that an increase in these factors tends to lead to higher excess returns. However, the UK EREITs also show a slight positive correlation with SMB, suggesting that smaller companies have higher returns than larger ones. Skewness and Kurtosis are slightly negatively related to EREITs' returns in both markets, suggesting that increased asymmetry and peakiness in return distributions are associated with lower returns.

For MREITs in the US, the only factor showing a significant positive correlation is Momentum, suggesting that MREITs that performed well in the past will continue to do so in the future. Conversely, Skewness has a slight negative correlation with MREITs' returns, indicating that increased asymmetry in returns can potentially lower the returns.

For HREITs in the UK, there is a significant positive correlation with excess market return, SMB, and Momentum, while Skewness and Kurtosis are slightly negatively related. In the US, the excess return of HREITs shows a significant positive correlation with the excess market return and Momentum. Interestingly, Kurtosis shows a slight positive correlation, indicating that a higher level of extreme values in return distribution can potentially increase HREITs' returns.

The R-squared values for all models are relatively high, indicating that the regression models explain a significant portion of the variability in the excess returns of the REIT types.

In summary, excess market return and Momentum appear to have a consistent positive impact on all types of REITs in both markets. SMB also has a positive impact on EREITs in the UK and HREITs in both markets. However, the impact of Skewness, Kurtosis, and HML varies across different types of REITs and markets. These insights can be beneficial for investors and financial managers by providing a clearer understanding of the factors influencing the performance of these REITs.”

### 5.5 Influence of COVID-19

The price of REITs fluctuates significantly under the impact of economic crises and epidemics. The REITs index shows a faster U-shape recovery than other equities, and the characteristics of high dividends make its overall yield relatively stable in the long term. In particular, as of the end of June 2021 in North America, the average annual total return of EREITs in the past 40 years had reached 11.51%, of which the annualized dividend return had reached 6.69%, contributing 58% of the total return. Over the past 20 years, the average annual total rate of return of EREITs has been 10.48%, of which the dividend yield is 4.85%, accounting for 46% of the total rate of return; comparatively, in the last 10 years, the average annual total return of equity REITs has been 10.29%, of which the dividend yield is 3.99%, which also contributes 39% of the total return [12].

Overall, the dividend income of EREITs in North America contributes 40–60% of their total income, on average. Among them, the historical average dividend yield of healthcare REITs is the highest. As of the end of June 2021, the dividend yield of the past 20 years was 6.45%, contributing 54.3% of the total return; independent store REITs and diversified REITs followed, with a dividend yield (past 20 years) of 6.16% and 5.48%, respectively; and the historical average dividend yield of other EREITs was mostly between 4% and 5% [12]. There are other new REITs, such as single-house rentals, forest farms, infrastructure, and data centers; however, due to their late launch time and the impact of interest rate reduction after the financial crisis, their historical average dividend yield is low at the present.

The stock price performance of most traditional REITs has been poor in the past five years, and dividend income has become the main, or even the total, source of income during this period. Among these, in the past five years, the total return of residential mortgage REITs, healthcare properties, hotels, shopping centers, community business centers, offices, and diversified REITs was almost all from the contribution of dividend income. The dividend income contribution ratio of REITs of independent stores, specially real estate, and CRE mortgage loans also reached more than 65%. Compared with the share price of industrial infrastructure and rental housing over the past 20 years, the dividend income contribution ratio has contributed less than 5% to the income of industrial infrastructure and logistics centers.

## 6 Discussion

The excess returns of the REITs of the five countries are positively and highly related to momentum. Previous researchers have had similar findings using different models in the different stock markets, noting more significant momentum effects in the developed market [49,50]. The effects of the coefficients of skewness and kurtosis of the five countries differ from momentum, and these two variables have a tiny impact on REIT excess returns. However, skewness has been proven highly relevant to future stock volatility, while kurtosis cannot explain volatility due to the unstable robust test [51]. Hence, the influence of these two variables on the excess returns of the REITs is uncertain because the coefficients for different countries are different. As such, Hypothesis 1 is supported: the momentum of the returns of the REITs is positively related to the excess returns of the REITs in the five countries. This finding is consistent with Lee et al. [52], that momentum is a factor positively influencing the return of the REIT market. However, Hypothesis 1 is not supported by the regression results, in that the momentum of the monthly returns of the REIT indexes is not always significantly related to the excess returns of the REIT indexes in the different countries. First, the absolute values of the coefficients of the momentum of the monthly returns of the REIT indexes of four of the countries (excluding the US) are small, indicating that the unit changes of the momentum of the monthly returns of the REIT indexes of these countries create only small changes to the excess returns of their REIT indexes. Under this condition, the impact of the momentum of the monthly returns of these national REIT indexes is limited. Second, combining the coefficients of all these independent variables, the absolute values of the momentum of the monthly returns are relatively small, except for the US and Canada. Therefore, compared to the absolute values of the coefficients of the other independent variables, the momentum of the monthly return of the REIT indexes does not play a dominant role in determining the values of the excess return of the REIT indexes. Thus, the conclusion cannot be made that the momentum of the monthly return of the REITs is significantly related to their excess return, so Hypothesis 1 is rejected.

Hypotheses 2 and 3 are also be rejected according to the calculation results of the regression analysis, where both the skewness and kurtosis of the monthly returns of the REITs are not significantly related to the excess returns of the national REIT indexes of the five countries. According to Table 4, the absolute values of the coefficients of skewness and kurtosis are very small, close to zero. These results suggest that the changes in the skewness and kurtosis of the monthly returns of these national REIT indexes create little influence on the excess returns of the monthly returns of these REIT indexes. Thus, it is rational to believe that the impact of skewness and kurtosis on the excess return of REITs is limited. These findings in relation to skewness are similar to the results of Antonacci [53] and Moss et al. [54], who found the less correlations between the annual return of REITs and skewness. We suggest two possible reasons for the negative relationship between REIT return and skewness in the US. First, the US has tighter connections among its domestic industries than other countries, so skewness may also connect with other economic aspects. Second, the scale of other countries' REITs is much smaller than that of the US. Thus, the skewness of the REITs does not play a significant role in influencing REIT returns.

We also observe that the regression results are similar for both the pre-COVID and COVID periods, and thus offer the following theories to explain the phenomenon. First, a pandemic's impact on financial markets differs from that of other contingencies such as wars, and global financial crises, which can destroy the capital and financial market; an epidemic only affects the economy in the short term. Jordà et al. [55] studied the economic activity during the 14th century Black Death and the 1918 Great Influenzas; they found that health workers created high productivity and got more payments because of aggressive monetary and financial policies increasing liquidity and demand. Thus, increasing liquidity can help the economic and financial market recover quickly, and even exceed their pre-pandemic level. Second, the Fama–French model explains the excess return with multiple variables, and the coefficient estimate converges when the trend of other risk factors is similar to the excess return. This study used the major indexes of the five countries' constituent stocks to build the risk factors, and the REIT markets share a similar U-shape recovery trend to that of their respective stock markets. This similar trend might explain the similar coefficient results.

Furthermore, the results of the EREITs, MREITs, and HREITs of the UK and the US suggest that the relationships between these REITs' excess returns and the individual variables, such as momentum, skewness, and kurtosis are not consistent. As for the EREIT, the regression models for the UK and the US show that the excess return of the EREITs has a positive relationship with momentum but not with skewness and kurtosis. Nevertheless, the results for the MREITs and HREITs suggest this is not so. According to the regression results for the HREITs for the UK sample, the relationships between the excess return of REITs and the three variables momentum, skewness, and kurtosis are similar to those for the EREITs. However, the regression results for the MREITs and HREITs based on the US sample show that the relationships between the excess return of REITs and the three examples is different—the return is positively related to momentum and kurtosis but negatively related to skewness. These results suggest that the excess returns for the three REIT types do not share the same relationship with these variables across the different countries. This phenomenon is also found in this study of REITs in these countries.

The next two points compare the absolute values of the momentum, skewness, and kurtosis for the three REIT types. First, momentum in the three REITs is more significant than skewness and kurtosis in determining the excess returns of these REITs in the UK and the US. Second, comparing the momentum coefficients for these two countries shows that momentum plays a more critical role in determining the excess returns of the EREITs and HREITs in the US than in the UK. However, findings are dissimilar for skewness and kurtosis, suggesting that these two variables do not play a significant role in determining the excess returns of the EREITs and HREITs, and that these results hold across different countries.

Comparing the coefficients of momentum, skewness, and kurtosis for the regression models of the composite REITs and the EREITs, MREITs, and HREITs for the UK and the US produces some exciting phenomena. The UK market show no significant differences between the coefficients in different REITs. However, this is not the case in the US market, where the coefficients of the three variables for the composite REITs are much more significant than those of the sub-REITs. Furthermore, the implications of skewness and kurtosis to the excess returns of the REITs have also changed in the sub-REITs market: the skewness becomes negatively related to the excess return of the REITs in the sub-REITs market while it is positive for the composite market, and the kurtosis becomes positive in the MREITs and HREITs markets while it is negative in the composite market. Hence, this reveals that the influence of momentum, skewness, and kurtosis on the composite REITs and the sub-REITs is inconsistent in the US.

The momentum, skewness, and kurtosis affect different REIT types. The market scale could be one of the reasons leading to this phenomenon. According to a report from NAREIT (2019), the US's total REIT market value reached $1.3 trillion, including $1.2 trillion in EREITs, $80 billion in MREITs, and the remainder in HREITs. This difference in REIT market share may cause a difference in the power of the risk factors' impacts on the various REIT types. The various impact of risk factors between EREITs and MREITs may be due to their internal characteristics. As mentioned previously, although an MREIT is a type of REIT, MREITs invest in mortgages, and REITs invest in real estate, so that MREITs face credit risk, not just interest risk. Thus, the two different investment styles may lead to the different risk factor impacts.

The last finding is an exciting one: although the characteristics and size of the different types of REITs can create various impacts for risk factors, the momentum impact on REITs and EREITs should not differ much from that of the EREITs, which are the most significant component of REITs, and also the most common type of REIT. This phenomenon warrants further study in future research.

This study provides significant insights for investors and policymakers alike into the behavior of different types of REITs across five different markets. It presents a comprehensive understanding of how market factors, firm characteristics, and risk metrics correlate with REIT returns. For investors, the study indicates that the excess market return, size, value, and momentum factors significantly impact REIT returns, thus shaping their portfolio diversification strategies. Particularly, adopting a momentum strategy seems profitable across all REIT types. However, negative correlations with skewness and kurtosis imply that asymmetry and extreme values in return distributions are generally associated with lower returns, indicating the importance of risk assessment. For policymakers, the study offers valuable insights into market dynamics and their effect on REITs, highlighting the need for regulations that maintain market stability, enhance transparency, and promote market efficiency. The significant influence of momentum factor underscores the need for timely information disclosure to avoid momentum-based price distortions. In essence, this study offers valuable guidance for optimizing investment decisions and regulatory measures in the REIT market.

## 7 Conclusion

COVID has significantly impacted the economic and financial market within the last two years, and REIT stocks show a solid capacity for risk-aversion. This study employed an extended version of the Fama–French model to identify how control variables and COVID affected REITs. The study analyzed the influence of five controlling variables—HML, SMB, momentum, skewness, and kurtosis—on the REITs' excess returns. We collected daily data from Australia, the UK, the US, Japan, and Canada and split it into three periods: full period, pre-COVID, and COVID. Then, we tested the how those variables affect the return of REITs in the different periods and across different types of REITs, namely EREITs, MREITs, and HREITs.

The study is the first to add skewness and kurtosis to the Fama–French five-factor model to test cross-national REIT return data. The combined results indicate that momentum is positively related to the excess return of the REITs for different situations, such as different types of REITs and different economic contexts. However, the influences of skewness and kurtosis are not consistent, and vary with the types of REITs and the economic context. The results show that momentum is more significant than skewness and kurtosis in affecting the excess return of the REITs. Our results differ from previous studies in that skewness shows more effect than kurtosis on REITs.

This study is the first to test the impact of COVID on REIT excess return. However, the empirical results shows that the effect of COVID is not significant in this model, which is consistent with the past literature [14,48], suggesting that pandemics cannot have a long-term impact on financial markets under financial and monetary policy interventions. We also believe the substantial risk resistance of REITs is the reason for this phenomenon.

Furthermore, aligned with previous literature, our study suggests that the REITs behave more like a stand-alone sector and less like normal stock. First, REITs have to pay dividends annually and at restricted from investing outside of real estate sectors; by contrast, normal companies can use dividends to invest in different industries. Second, the average turnover ratio of stocks is 115% higher than REIT stocks, and the number of financial analysts in REIT companies is significantly less than that in other stock companies [56]. Third, the kurtosis and skewness consistently affect normal stock [57], but this is not the case in our study.

Finally, in comparing the results of EREITs, HREITs, and composite REITs in the UK market, this research finds that the influences of momentum, skewness, and kurtosis are consistent in this REIT market. Nevertheless, this is not the case when applied to different types of REITs in the US market, which showed that the implications and the significance of momentum, skewness, and kurtosis for the returns of these types of REITs in the US are not consistent. Hence, it can be concluded that momentum positively influences the returns of the REITs, but that the influence of skewness and kurtosis is uncertain. In future research on REITs, there are still many aspects to explore in this area. One direction is to examine different segments of REITs, such as hotel REITs, factory REITs and residential REITs. Another potential direction is to investigate the effect of other factors such as asymmetric factors and macroeconomic factors.

## Author contribution statement

Wendi zhang: Conceived and designed the experiments; Performed the experiments; Analyzed and interpreted the data; Contributed reagents, materials, analysis tools or data; Wrote the paper.

Bin Li: Conceived and designed the experiments; Analyzed and interpreted the data; Wrote the paper.

Eduardo Roca: Conceived and designed the experiments; Wrote the paper.

## Data availability statement

Data will be made available on request.

## Declaration of competing interest

The authors declare that they have no known competing financial interests or personal relationships that could have appeared to influence the work reported in this paper.

## Footnotes

1 The 2008 GF C is believed to have started in 2007; hence, 2007 is regarded as the dividing line here.
